# Author Response to Kleebayoon and Wiwanitkit

**DOI:** 10.1093/ptj/pzaf054

**Published:** 2025-04-24

**Authors:** Yoav Gimmon, Yael Arbel, Liora Shmueli

**Affiliations:** Department of Physical Therapy, Faculty of Social Welfare & Health Studies, University of Haifa, Haifa, Israel; Department of Otolaryngology–Head and Neck Surgery, Sheba Medical Center, Tel Hashomer, Israel; Department of Management, Bar-Ilan University, Ramat Gan 52900, Israel; Clalit Health Services Ringgold standard institution, Physiotherapy, Tel Aviv, Israel; Department of Management, Bar-Ilan University, Ramat Gan 52900, Israel

We appreciate the opportunity to respond to the letter^1^ addressing our article, *“*Evaluating the Potential of Large Language Models for Vestibular Rehabilitation Education: A Comparison of ChatGPT, Google Gemini, and Clinicians.”^2^ The letter raises several insightful points, and we welcome the chance to clarify aspects of our study. Our research aimed to assess the capabilities of large language models (LLMs) in vestibular rehabilitation education, particularly in comparison to human expertise. By systematically evaluating their performance in factual knowledge and clinical reasoning, we sought to explore both the potential benefits and inherent limitations of artificial intelligence (AI)-driven tools in medical education and practice.

In our study, we utilized 2 human groups and 2 large language models (LLMs)—ChatGPT and Google Gemini. We did not compare the human groups to each other, as this was not the aim of our study. The human groups (experienced vestibular physical therapists and students prior to in-depth education on the vestibular system) were included to highlight the differences between factual knowledge and clinical reasoning. Indeed, our findings reflected this: while the LLMs excelled in answering factual knowledge questions, they performed worse than experienced physical therapists in areas requiring clinical reasoning. However, they outperformed the inexperienced students who lacked clinical knowledge.

We sampled 30 participants from each human group and performed *t* tests to compare performance. This sample size was sufficient for comparing performance in factual knowledge and clinical reasoning. However, we acknowledge that for exploring broader constructs such as attitudes toward the use of AI or perceptions of its utility, would require a larger sample size and a different sampling method to ensure robust statistical results and meaningful interpretations.

An important aspect is the potential of LLMs as educational tools. Our findings indicate that the models relied more on the volume of information available online rather than on its quality and up-to-dateness. If LLMs improve their ability to prioritize recent scientific advancements over outdated but more widely available information, they could become more reliable sources of knowledge. This discrepancy was particularly evident in our study when examining treatment recommendations following benign paroxysmal positional vertigo.

We believe that AI tools, such as ChatGPT and Google Gemini, can be used effectively by clinicians if approached wisely. To ensure the accuracy and safety of AI recommendations, we propose integrating AI bot-based tools that are trained on evidence-based clinical guidelines, which are continuously updated. These guidelines, derived from rigorous research and expert consensus, can help clinicians use AI tools in alignment with current best practices. However, it is important to acknowledge that AI cannot fully replicate the complexity of clinical reasoning. Clinical reasoning is a multifaceted process that integrates knowledge, experience, and sensitivity to both verbal and nonverbal cues during patient interactions. It is essential to acknowledge the limitations of LLMs, which are text-based and thus inherently limited in their ability to replicate the full scope of human clinical reasoning.

Future research is essential to fully explore these capabilities. Expanding studies to include larger and more diverse populations worldwide will allow us to refine the capabilities of LLMs in various medical and niche fields while also improving their ability to recognize cultural differences. As AI continues to evolve, studying its application across diverse populations will help refine its role in clinical education and practice.

In conclusion, while large language models demonstrate remarkable capabilities in factual knowledge retrieval and structured information delivery, they remain inherently limited in replicating the nuanced clinical reasoning essential for patient care. Human expertise—shaped by experience, critical thinking, and interpersonal skills—remains indispensable in medical practice. However, with ongoing advancements, AI-powered tools have the potential to enhance clinical education, support decision-making, and bridge knowledge gaps, provided they are used judiciously and in alignment with evidence-based guidelines. The challenge now lies in harnessing the strength of AI while mitigating its limitations. Future research must focus on refining these models for clinical applications, ensuring they are grounded in current best practices and ethical considerations. By developing AI to complement—not replace—clinicians, we can create a future where technology enhances learning, informs decision-making, and ultimately improves patient outcomes.
